# Phospholipase A2 Group IIA Is Associated with Inflammatory Hepatocellular Adenoma

**DOI:** 10.3390/cancers16010159

**Published:** 2023-12-28

**Authors:** Sadahiro Iwabuchi, Kenta Takahashi, Kazunori Kawaguchi, Akihisa Nagatsu, Tadashi Imafuku, Shigeyuki Shichino, Kouji Matsushima, Akinobu Taketomi, Masao Honda, Shinichi Hashimoto

**Affiliations:** 1 Department of Molecular Pathophysiology, Institute of Advanced Medicine, Wakayama Medical University, Wakayama, Wakayama 641-0011, Japan; iwabuchi@wakayama-med.ac.jp (S.I.);; 2 Department of Human Pathology, Graduate School of Medicine, Kanazawa University, Ishikawa, Kanazawa 920-0934, Japan; 3 Department of Gastroenterology, Kanazawa University Hospital, Ishikawa, Kanazawa 920-0934, Japan; 4 Department of Gastroenterological Surgery I, Hokkaido University Graduate School of Medicine, Hokkaido, Sapporo 060-8648, Japan; 5 Division of Molecular Regulation of Inflammatory and Immune Disease, Research Institute for Biomedical Sciences, Tokyo University of Science, Chiba, Noda 278-8510, Japan

**Keywords:** hepatocellular adenoma, hepatocellular carcinoma, Pla2g2a, single-cell RNA sequencing

## Abstract

**Simple Summary:**

Phospholipase A2 Group IIA (PLA2G2A), discovered by single-cell gene expression analysis, is useful for the diagnosis of hepatocellular adenoma (HCA) with conventional HCA-specific markers. Improved diagnostic criteria will avoid unnecessary surgery and lead to options such as drug therapy.

**Abstract:**

Although benign hepatocellular adenomas (HCA) are very rare, recent observations have shown their occurrence in patients with diabetes mellitus. Consequently, most of these cases are treated by resection due to concerns regarding their potential progression to hepatocarcinoma (HCC). This decision is largely driven by the limited number of studies on HCC subtyping and the lack of molecular and biological insights into the carcinogenic potential of benign tumors. This study aimed to comprehensively investigate the subtype classification of HCA and to compare and analyze gene expression profiling between HCA and HCC tissues. One fresh inflammatory HCA (I-HCA), three non-B non-C HCCs, two hepatitis B virus-HCCs, and one normal liver tissue sample were subjected to single-cell RNA sequencing (scRNA-seq). Comparative analysis of scRNA-seq among different tissues showed that phospholipase A2 group IIA (*PLA2G2A*) mRNA was specifically expressed in I-HCA, following RNA-seq analysis in formalin-fixed paraffin-embedded tissues from other HCAs. Immunohistochemistry using the PLA2G2A antibody in these tissues indicated that the positive reaction was mainly observed in hepatocytes of I-HCAs and stromal cells surrounding the tumor tissue in HCC were also stained. According to a clinical database, PLA2G2A expression in HCC does not correlate with poor prognosis. This finding may potentially help develop a new definition for I-HCA, resulting in a significant clinical contribution, but it requires validation with other fresh HCA samples.

## 1. Introduction

The incidence of hepatocellular adenoma (HCA), a rare benign liver tumor, has become markedly increased in men owing to the growing prevalence of obesity and metabolic syndrome [1]. Although benign, HCAs may act as precursors to hepatocellular carcinoma (HCC) in metabolic syndrome. In contrast to other benign liver tumors, elective resection is recommended for all men with HCA in Japan, although little is known about the potential progression to HCC. In women, tumor size >5 cm is closely correlated with risk factors for malignant transformation and hemorrhage [2,3].

Results of comprehensive molecular and immune–histological studies aided representative HCA subtype classification as follows: hepatocyte nuclear factor 1 homeobox A (HNF1A) inactivated (H-HCA), inflammatory HCA (I-HCA), β-catenin mutated (β-HCA or β-IHCA), and sonic hedgehog activated (sh-HCA) [1,3,4,5,6]. The majority of HCA subtypes are I-HCA (33%), with strong and diffuse expression of serum amyloid A (SAA) and C-reactive protein (CRP) observed in the tumors; however, some tumors with these positive reactions are difficult to clearly distinguish from HCC. Detailed clinical data analysis shows that approximately 3% of HCA patients develop HCC and that 7% of HCAs share intermediate histological features with HCC [4]. Therefore, diagnosing and distinguishing between HCC and different types of HCA based on imaging findings alone can prove challenging, making biopsy with genomic analysis and careful pathological examination, including immunohistochemistry for I-HCA markers, prerequisites for accurate diagnosis [4]. Patients with HCA should be appropriately treated in accordance with subgroup classification to avoid fatal complications, such as malignant transformation. Considering the rarity and potential malignant transformation of HCA in the Japanese population, understanding the characteristics of and detailed gene expression dynamics in HCA tissues has the potential to advance the evolution of its treatment approach, which currently relies solely on resection for a spontaneous cure. However, to the best of our knowledge, no comprehensive gene expression analysis has been conducted using fresh HCA tissues. 

Here, we performed single-cell RNA sequencing (scRNA-seq) on fresh I-HCA tissue and analyzed I-HCA alone, normal liver, and different types of HCCs to detect specific gene expression features in I-HCA.

## 2. Materials and Methods

### 2.1. Characteristics of the Patient

The study included nine patients: three suspected HCA, three with non-B non-C-HCC (NBNC-HCC), two with hepatitis B virus-derived HCC (HBV-HCC), and one with hepatitis C-derived HCC (HCV-HCC). Informed consent was obtained from all patients for participation in the present research and publication of the results. Here, we present only the detailed medical records of patients with HCA whose tissues were analyzed by scRNA-seq. A 33-year-old man with suspected HCC carcinoma and a normal liver background was admitted to the hospital. He had no smoking history, rarely drank alcohol, had no diabetes, and had a BMI of 22.9. Contrast-enhanced computed tomography (CT) and magnetic resonance imaging (MRI) revealed a mass with a maximum diameter of 60 mm without obvious vascular infiltration. This is atypical of I-HCA. Surgical resection was performed promptly and the specimens were collected. Subsequent histopathological diagnosis revealed SAA- and CRP-positive tissues.

### 2.2. Human Liver Tissue Dissociation and Single-Cell RNA Sequencing (scRNA-seq)

The detailed methods were described in the Supplementary Document. In brief, the samples were minced using biological tweezers and the pieces were collected in a centrifuge tube with cold Hanks’ balanced salt solution (HBSS) containing 5% fetal bovine serum (FBS). After centrifugation, the supernatant was discarded and replaced with the warmed 10 mL Roswell Park Memorial Institute (RPMI) 1640 Medium (Thermo Fisher Scientific, Waltham, MA, USA) with 5% FBS and 500 μg of Liberase TH (Roche, Basel, Switzerland); then, it was incubated with gentle shaking (300–400 rpm) at 37 °C for 30 min. Next, 500 μg of DNaseI (Qiagen, Düsseldorf, Germany) was added to the solution with gentle shaking at 37 °C for 10 min. The entire mixture was filtered using a 100 μm Cell Strainer with gentle mashing using a piston for an injection cylinder. The supernatant was replaced with 4.8 mL of fresh cold RPMI medium in each tube. A 20% Percoll solution (Cytiva, Marlborough, MS, USA) with cell solution was added to the 60% Percoll solution and then centrifuged. An intermediate layer with target cells was carefully moved and the isolated cell solution was filtered using 40 μm Flowmi™ Tip Strainers (Bel-Art-SP Scienceware, Wayne, NJ, USA). We used an Nx1-seq (next-generation 1-cell sequencing) device for scRNA-seq [7]. The protocol for preparing barcoded beads was slightly modified from that described in the instruction manual for the GS Junior Titanium emPCR Kit (Lib-B) (Roche, Basel, Switzerland) [8]. The detailed methods for making a sequencing library were described in the supplemental data. High-throughput sequencing was performed on samples with a 20% PhiX control using the MiniSeq High Output Kit (Illumina Inc., San Diego, CA, USA), 150 cycles pair-end, 25/125 or 75/75 cycles), or NextSeq 500/550 High Output Kit v2.5 (Illumina, 150 cycles pair-end, 25/125 or 75/75 cycles). Pair-end FASTQ files were mapped and annotated using bowtie 2 software v2.2.26 and Perl custom scripts [8]. The sequences were aligned against RefSeq mRNA (ftp://ftp.ncbi.nih.gov/refseq/H_sapiens/mRNA_Prot/, accessed on 19 December 2023) as a reference sequence. After mapping, the barcode was linked to its paired read 2 alignment data and the genes were counted for each barcode. Cell clustering was performed by t-distributed Stochastic Neighbor Embedding (tSNE) analysis using Seurat v2.3.0, on R-3.6.3. The raw fastq files of scRNA-seq data were deposited in the DDBJ website (https://ddbj.nig.ac.jp/search (accessed on 19 December 2023)), DRA017022 (DRR500608-DRR500611).

### 2.3. Immunohistochemistry (IHC)

In accordance with standard methods, formalin-fixed paraffin-embedded (FFPE) tissues were prepared by fixing fresh tissues using 4% paraformaldehyde at 4 °C and embedding them in paraffin. Tissue sections (4 µm thick) were cut, dewaxed, and rehydrated using xylene and graded alcohol. Hematoxylin and eosin (H&E) staining was performed. The sections were then inactivated by treating them with an antigen activator (citric acid pH 6.0) for 20 min at 95 °C and they were incubated with anti-human PLA2G2A polyclonal antibody (1:200 dilution, PA5-102403, Thermo) or anti-human SAA1 + SAA2 (1:150 dilution, PA5-102456, Invitrogen, Waltham, MA, USA) overnight at 4 °C. Thereafter, the sections were treated with rabbit anti-IgG antibodies for 30 min at 20 °C and visualized following treatment with 3,3-diaminobenzidine for 10 min at 20 °C. Subsequently, the sections were counterstained with hematoxylin. All stained sections were examined under a fluorescence microscope (BZ-X710; KEYENCE, Osaka, Japan).

### 2.4. RNA Sequencing (RNA-seq)

The detailed methods were described in the Supplementary Document. Total RNA from the FFPE block was obtained using a NucleoSpin total RNA FFPE kit (Marcherey-Nagel GmbH and Co. KG, Düren, Germany) according to the manufacturer’s instructions. RNA quality was evaluated using an Agilent 4200 TapeStation (Agilent Technologies Inc., Santa Clara, CA, USA) and the RNA concentration was measured using a Qubit Fluorometer (Thermo). A total of 1000–3500 ng of RNA from each sample was used and libraries for sequencing were constructed using TruSeq Stranded mRNA (Illumina) according to the manufacturer’s protocol. High-throughput sequencing of the samples was performed using a NextSeq 500/550 High Output Kit v2.5 (Illumina, 75 cycles pair-end, 40/40 cycles). The bulk RNA-Seq results were analyzed using the CLC Genomics Workbench Version 12.0.2 (Filgen Inc., Nagoya, Japan). The raw transcript per million (TPM) data obtained from RNA-seq are provided in Supplementary Data S1 and S2. Target gene sets were analyzed using the Gene Ontology enrichment analysis tool Metascape [9].

## 3. Results

### 3.1. scRNA-seq of HCA Sample

**Figure 1:**
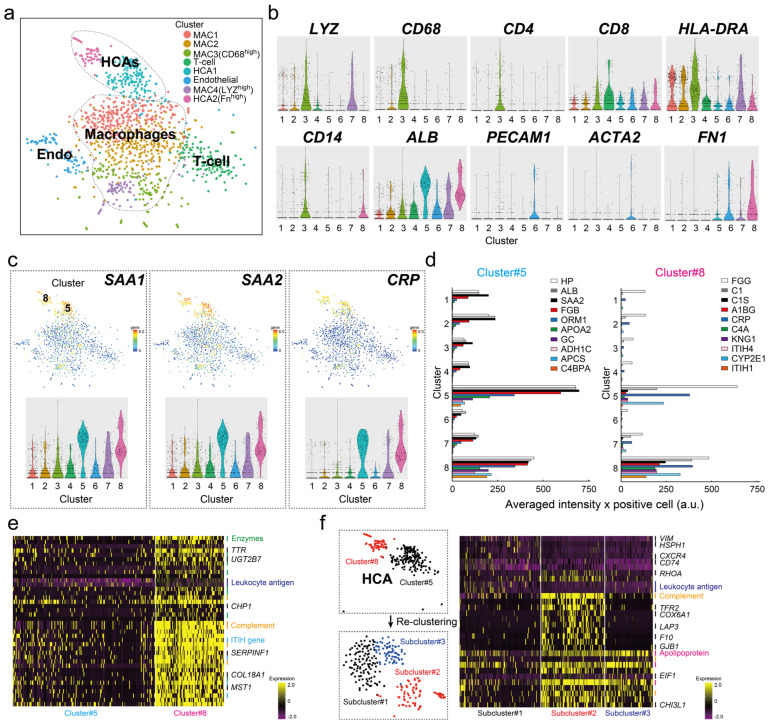
scRNAseq of HCA sample. (**a**) The tSNE plot of HCA cell clusters based on 1591 cells. It was classified into eight cell clusters; four macrophages (cluster #1, #2, #3, and #7 MAC1MAC4), two HCA (cluster #5 HCA1, #8 HCA2), one T-cell (cluster #4), and one endothelial (Endo, cluster #6). (**b**) Violin plots for representative 10 cell marker genes (gene symbol). (**c**) Feature plot and violin plot for conventional specific inflammatory HCA (IHCA) markers; *SAA1*, *SAA2*, and *CRP*. “5” or “8” means the cluster # shown in (**a**). (**d**) The bar graphs of the distribution for the top 10 specific genes determined to cluster #5 and cluster #8 through all clusters. The gene symbol corresponding to each color-coded bar graph is presented in the upper right corner of each graph. The *x*-axis shows averaged intensity × positive cell (a.u.) and the *y*-axis shows the cluster number. (**e**) Differential expressed genes (DEGs) between cluster #5 and cluster #8. The color in heatmap from yellow to violet reveals the gradual expression intensity differences from high to low. The letters to be listed on the right side of the heatmap indicate the representative gene groups or gene symbols. For example, there are eight “Enzymes” (green lines), such as *CYP2C9*, *LDHA*, and *CYB5R3*; two “Leukocyte antigen” (blue lines); five “Complement” (orange lines); and four “ITIH gene family” (sky blue lines). (**f**) Reclustering of two hepatocellular cell clusters #5 and #8. The heatmap shows the differential expressed genes among three subclusters. Additionally, there are three “Apolipoprotein” (pink lines) groups.

We performed scRNA-seq of liver tissue from a fresh I-HCA sample and a total of 1591 cells were classified into eight cell types, namely, two albumin (*ALB*)-highly positive hepatocyte cell clusters [cluster number(#)5 HCA1, #8 HCA2 in Figure 1a,b], four macrophages (#1, #2, #3, #7 MAC1-MAC4 clusters), one plated and endothelial cell adhesion molecule 1 (*PECAM1*)-positive endothelial cell (EC, cluster #6), and one T-cell (cluster #4). The expression of the I-HCA-specific markers *SAA1*, *SAA2*, and *CRP* were high in both hepatocyte clusters (#5 and #8), indicating that the specific genes determining both cell populations were not related to conventional I-HCA markers (Figure 1c). Gene expression associated with other HCA subtypes and HCC marker genes were also not involved in the determination of either cluster (Supplementary Figure S1). Figure 1d indicates that the expression profiles of the top 10 marker genes characteristic of the HCA cluster #5 showed that some genes, such as hepatoglobin (*HP*), *ALB*, and *SAA2* expressed in all clusters, with different expression intensities. The expression intensity of *CRP* specifically detected in cluster #8 was identical to that detected in cluster#5 but the complement-related genes (*C1S* and *C4A*) and the inter-alpha-trypsin inhibitor heavy chain (*ITIH*) gene family tended to be higher in cluster #8 (Figure 1d). The comparative analysis of the averaged gene expression profiles of all cells in clusters #5 and #8 revealed that not only complement-related genes and the *ITIH* gene family but also serpin family F member 1 (*SERPINF1*), collagen type XVIII a1 chain (*COL18A1*), and macrophage stimulating 1 (*MST1*) tended to be highly expressed in cluster #8 (Figure 1e). We performed a re-clustering of the two hepatocellular cell clusters, which were divided into three subclusters, as shown in Figure 1f. Each subcluster was distinguished by several characteristic genes, particularly fibroblast growth factor receptor 2 (*FGR2*) and cytochrome C oxidase subunit 6A1 (*COX6A1*), which were highly expressed in subcluster2. Thus, various gene expression analyses obtained by scRNA-seq of a single HCA sample were performed, which revealed the presence of different hepatocyte cell populations with different gene expressions. However, our target gene Phospholipase A2 group IIA *(PLA2G2A)* was not specifically extracted as an I-HCA marker by scRNA-seq of only one fresh HCA sample.

### 3.2. Comparison with Other HCC Samples by scRNA-seq Analysis

**Figure 2:**
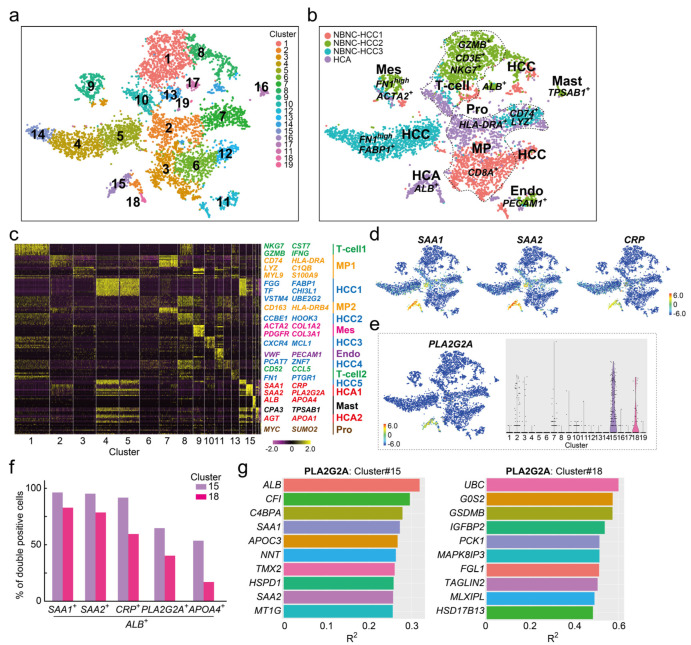
scRNAseq of one HCA and three NBNC-HCC samples. (**a**) The tSNE plot of the total for 7612 cells (NBNCHCC1; 1897 cells, NBNCHCC2; 2001 cells, NBNCHCC3; 2123 cells, and HCA; 1591 cells). The merged cells were divided into 19 clusters. (**b**) Cell types and sample-identified tSNE plots. The majority of the cells in NBNCHCC1 (red), NBNCHCC2 (green), and NBNCHCC3 (sky blue) were *CD8A*^+^ macrophages (MP, cluster #3, #6), *GZMB*^+^*NKG7*^+^ Tcell (cluster #1), or *FN1*^high^*FABP1*^+^cancer cells (clusters #4 and #5). (**c**) Heat map analysis of the four samples. The color in the heatmap from yellow to violet reveals gradual differences in expression intensity from high to low. The letters listed on the right side of the heatmap indicate representative gene groups or gene symbols. MP1, macrophage cluster 1; Mes, mesenchymal cells; Endo, endothelial cells; Mast: Mast cells; and Pro, MYC^high^ proliferated cells. (**d**) Feature plots of representative I-HCA makers, *SAA1*, *SAA2*, and *CRP*. The color bar, from red to blue through yellow, reveals gradual expression intensity differences from high to low through the middle. (**e**) *PLA2G2A* features and violin plots. (**f**) Ratio of double-positive cells to *ALB* and each gene for total cells in HCA clusters #15 or #18. Violet bars: Cluster #15; red bars: Cluster #18. (**g**) Gene–gene correlation plots associated with *PLA2G2A* in clusters #15 and #18. The *x*-axis indicates the coefficient of determination (R^2^).

To identify HCA-specific cell populations in more detail, scRNA-seq analysis was performed on three non-B and non-C HCC (NBNC-HCC) samples for comparison. A total of 7612 cells (NBNC-HCC1; 1897 cells, NBNC-HCC2; 2001 cells, NBNC-HCC3; 2123 cells, HCA; 1591 cells) were classified into 19 cell clusters; five cancer cells (#4, #5, #8, #12, and #14), four macrophages (clusters #2, #3, #6, and #7), three T-cells (#1, #10, and #13), two HCA cells (#15 and #18), one hepatocyte (#17), one EC (#11), one mesenchymal cell (Mes, #9), one mast cell (Mast, #16), and one proliferated cell (Pro, #19) (Figure 2a,b). All samples had common T-cell (#1), Endo (#11), and Mes (#9) cell clusters but NBNC-HCC2 had fewer macrophage cell populations (green in Figure 2b) and NBNC-HCC3 had no distinguishable hepatocyte populations (“sky blue” in Figure 2b). The cancer cell clusters in NBNC-HCC3 cells were recognized as three clumps (clusters #4, #5, and #14) and most of the cells expressed a fibrosis marker *Fn1* strongly. When the NBNC-HCC3 tissue was digested into a single cell, the tumor had the most pronounced tendency for gross fibrosis. *PLA2G2A* was extracted as one of the top 10 genes for determining the HCA1 cluster (clusters #15 and #18) and other I-HCA markers *SAA1*, *SAA2*, and *CRP* (Figure 2c). Compared to the three novel I-HCA markers, the feature and violin plots clearly demonstrated that *PLA2G2A* was expressed specifically in HCA cell clusters (clusters #15 and #18) (Figure 2d,e). The ratios (%) of *PLA2G2A*^+^*ALB*^+^ hepatocytes to total cells in clusters #15 and #18 were 66.2% and 41.3%, respectively, indicating that not all cells in each cluster expressed *PLA2G2A* (Figure 2f). In contrast, the ratios of *SAA1*^+^*ALB*^+^, *SAA2*^+^*ALB*^+^, and *CRP*^+^*ALB*^+^ hepatocytes in cluster#15 were 98.5%, 97.4%, and 93.8%, respectively. Since the PLA2G2A expression in hepatocytes of I-HCA tissue was lower than three conventional HCA marker genes, it was possible that PLA2G2A was not extracted as a specific gene by scRNA-seq as shown in Figure 1. Gene–gene correlation analysis of *PLA2G2A* in HCA cluster #15 revealed that *ALB*, *SAA1*, and *SAA2* were the top 10 correlated genes; however, the coefficients of determination (R^2^) were not high (Figure 2g). In contrast, three genes; ubiquitin C (*UBC*), G0/G1 switch 2 (*G0S2*), and gasdermin B (*GSDMB*), had higher R^2^ (>0.5) correlations with *PLA2G2A* in cluster 18. Another I-HCA-specific candidate gene, apolipoprotein A4 (*APOA4*), was also detected in the heat map analysis of cluster #15 (Figure 2c). The expression of *APOA4* was specific to cluster #15 rather than to cluster #18 (Supplementary Figure S2a) and *ALB*^+^*APOA4*^+^ cells were also present (Figure 2f). Gene–gene correlation analysis for *APOA4* in cluster #15 showed that *G0S2* was the most highly correlated gene (R^2^ < 0.5) (Supplementary Figure S2b). As described above, *PLA2G2A* and *APOA4*, which were not extracted by scRNA-seq in I-HCA alone, were clearly identified as hepatocyte specific genes in I-HCA compared with other NBNC-HCC samples.

### 3.3. IHC of PLA2G2A in I-HCA Sample

**Figure 3:**
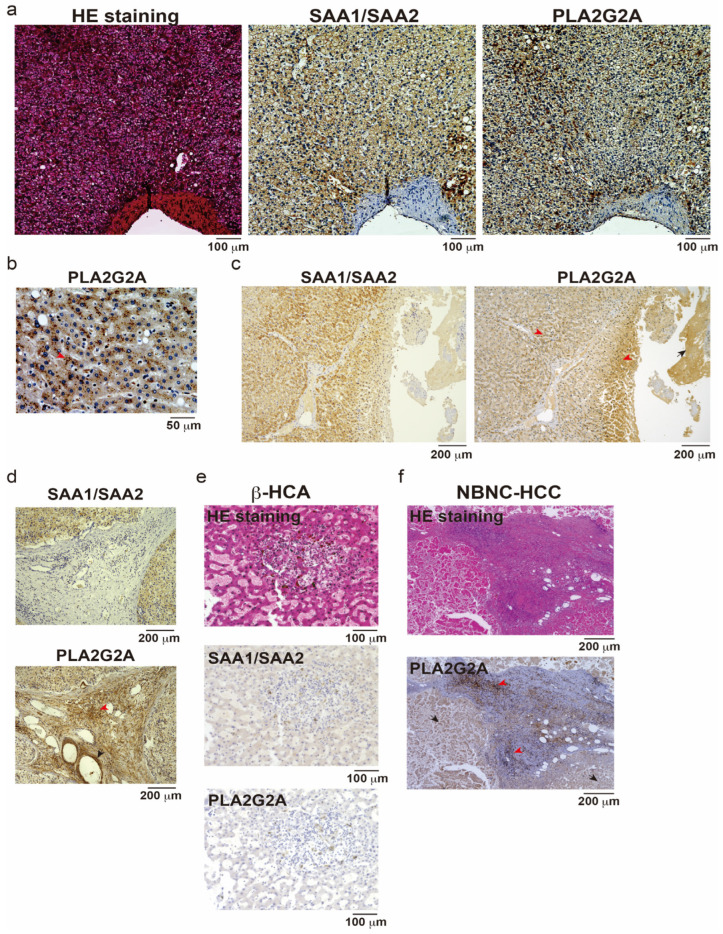
Immunohistochemistry (IHC) of three different IHCA, one bICA, and NBNC-HCC samples. (**a**) Hematoxylin and eosin (HE) staining, IHC for SAA1/SAA2 or PLA2G2A were performed on the IHCA sample. Scale bar = 100 μm. (**b**) IHC for PLA2G2A with high magnification. Red arrow shows a representative dotlike staining pattern of PLA2G2A. Scale bar = 50 μm. (**c**) Low magnification of IHC for SAA1/SAA2 or PLA2G2A in other IHCA tissue. Red arrows; positive reaction, black arrow; negative reaction. Scale bar = 200 μm. (**d**) IHC for SAA1/SAA2 and PLA2G2A in another IHCA sample. Red arrow; positive reaction, black arrow; negative reaction. Scale bar = 200 μm. (**e**) HE staining, IHC for SAA1/SAA2, or PLA2G2A were performed on the βHCA sample. Scale bar = 100 μm. (**f**) HE staining and IHC for PLA2G2A in NBNCHCC tissue. Red or black arrows; representative positive or negative staining. Scale bar = 200 μm.

To confirm whether the protein level of PLA2G2A was also upregulated in I-HCA samples, IHC analysis using anti-human PLA2G2A antibody was performed in three different I-HCAs and one β-HCA. SAA1/SAA2-positive and PLA2G2A-positive reactions were observed in similar regions but were not completely identical (Figure 3a). The PLA2G2A, stained as dots in the cytoplasm of hepatocytes, was observed with a few positive reactions in the nucleus (Figure 3b, red arrow). The anti-PLA2G2A antibody used for this staining also showed nonspecific reactions (Figure 3c, black arrow); however, the positive region was more localized than that of SAA1/SAA2. One I-HCA specimen showed that the PLA2G2A-positive reaction was not detected in stromal cells, including vascular endothelial cells and fibroblasts (Figure 3d, red or black arrows indicate positive or negative reactions, respectively). In contrast, SAA1/SAA2 and PLA2G2A reactions were negative in β-HCA samples (Figure 3e). In the NBNC-HCC specimens, PLA2G2A was stained non-specifically in the center of the tumor owing to necrosis (Figure 3f, black arrows). However, a positive reaction was regularly observed in the interstitial tissues of the tumor periphery (red arrows). One possibility is the oncogenic transformation of PLA2G2A-positive I-HCAs in this tumor tissue. Although *PLA2G2A* mRNA expression was mainly restricted to hepatocytes in the scRNA-seq analysis, it is possible that insufficient stromal cells were collected during the single-cell dispersion process. In addition, the PLA2G2A primary antibody used in this study is a commercial product and its specificity needs to be optimized using tissue fixation and inactivation methods.

### 3.4. Distribution of PLA2G2A Expression in Normal and HBV-HCC Samples

**Figure 4:**
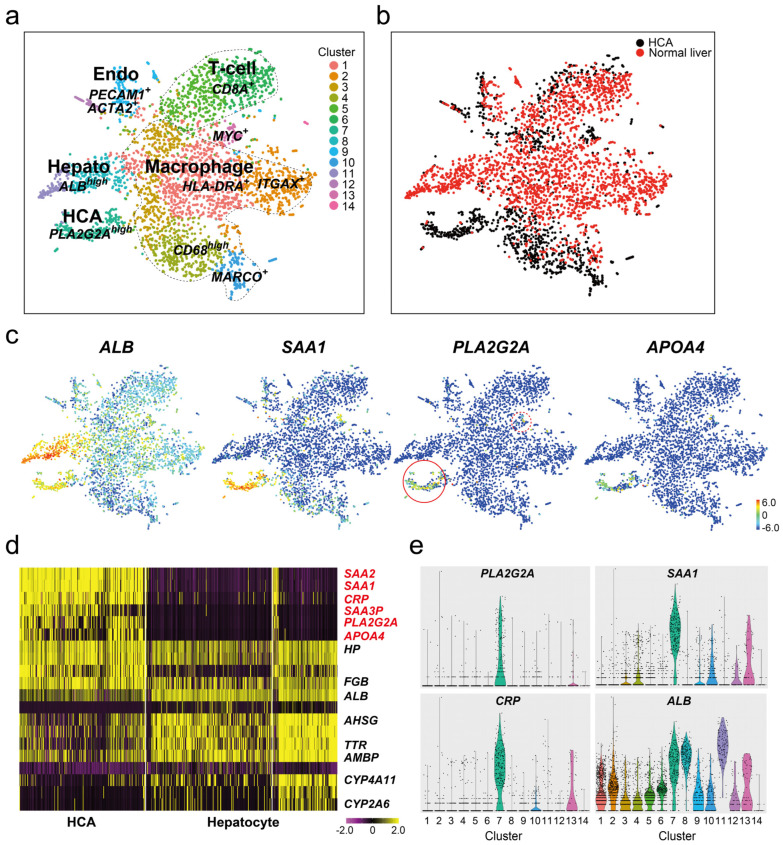
scRNA-seq of normal and adenoma tissue from one IHCA sample. (**a**,**b**) The tSNE plot of the total of 4292 cells (HCA; 1601 cells, normal liver; 2691 cells). Merged cells were divided into 14 cell clusters. The majority of cells in normal liver (red) was *HLA-DRA*^+^ or *ITGAX*^+^ macrophages (cluster #1 and #2), *CD8A*^+^ T-cells (cluster #5 and #6,) or *ALB*^high^ hepatocyte cell clusters (cluster #4 and #5). Black dots: adenoma (HCA), red dots: normal liver. (**c**) Feature gene expression plots of *ALB*, *SAA1*, *PLA2G2A*, and *APOA4* genes. The color bar indicates the intensity of gene expression from high (red) to low (blue) through the middle (green). Red circle: higher *PLA2G2A*^+^ cells and red dots circle: a weaker *PLA2G2A*^+^ cells. (**d**) The heat map analysis of adenoma (HCA, cluster #7) and normal liver (Hepatocyte, cluster #4 + cluster #5). The color in the heatmap from yellow to violet reveals the gradual expression intensity differences from high to low. The letters to be listed on the right side of the heatmap indicate the representative gene groups or gene symbols. Six representative genes shown in red color are I-HCA specific genes. (**e**) Violin plots of *PLA2G2A*, *SAA1*, *CRP*, and *ALB* genes. *x*-axis; clusters, *y*-axis; the intensity of gene expression (arbitral unit).

The expression of *PLA2G2A* mRNA in the normal liver and HBV-HCCs was examined using scRNA-seq analysis. A comparison of tSNE analysis between normal tissues and adenoma tissue in the same I-HCA patient showed 14 cell populations; these were common in *CD68*^high^ or *MARCO*^+^ macrophages (clusters #4 and #10), *PECAM1*^+^ vascular endothelial cells (cluster #9), *CD8A*^+^ T cells (cluster#5 and #6), and *MYC*^+^ proliferating cells (cluster #13) (Figure 4a,b). In contrast, the *ALB*^+^ hepatocyte population differed completely between normal tissues (clusters #9 and #12) and adenomas (cluster #7). *HLA-DRA*^+^ (cluster #1)-and *ITGAX*^+^ (cluster #2)-positive macrophage populations were prominent in normal tissues. The feature plots of *SAA1*, *PLA2G2A*, and *APOA4* showed that *ALB*^+^ hepatocytes in normal tissues were not strongly positive. Some *SAA1*^+^*CD68*^high^ and *SAA1*^+^*MARCO*^+^ macrophages (clusters #4 and #10) were detected in both tissues, confirming that *SAA1* was not specifically expressed in the hepatocytes of I-HCA tissues (Figure 4c). Six genes (*SAA2*, *SAA1*, *CRP*, *SAA3P*, *PLA2G2A*, and *APOA4*) listed in the heat map also indicated that the expression of *PLA2G2A* was limited to the adenoma tissue (Figure 4d), following the violin plot results (Figure 4e). *PLA2G2A* mRNA was highly expressed in cluster #7 (HCA) and less highly expressed in cluster #13 (*MYC*^+^ proliferated cells), whereas *SAA1* was found in several cell populations and few *CRP*-positive cells were detected in cluster #10 (*MARCO*^+^ macrophages).

**Figure 5:**
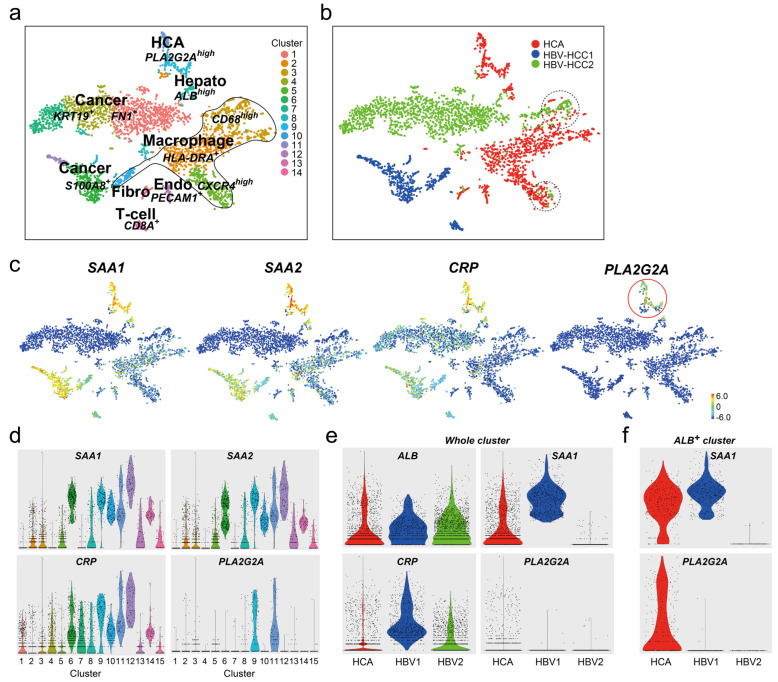
scRNA-seq of two HBV-HCC samples. (**a**,**b**) The t-SNE plot of total of 3900 cells (HCA; 1601 cells, HBV-HCC1; 631 cells, or HBV-HCC2; 1668 cells). Merged cells were divided into 14 cell populations: five cancer cells, four macrophages, one hepatocyte, one adenoma, one T-cell, one endothelial cell (Endo), and one fibroblast (Fibro). Red dots in adenoma (HCA), blue dots: HBV-HCC1, green dots: HBV-HCC2 sample. (**c**) Feature gene expression plots of *SAA1*, *SAA2*, *CRP*, and *PLA2G2A* genes. The color bar indicates the intensity of gene expression from high (red) to low (blue) through the middle (green). Red circle: higher *PLA2G2A*^+^ cells. (**d**) The violin plots (gene expression plot) of *SAA1*, *SAA2*, *CRP*, and *PLA2G2A* genes in each cell cluster. (**e**) The violin plots of *ALB*, *SAA1*, *CRP*, and *PLA2G2A* genes in each sample. Red: I-HCA, blue: HBV-HCC1, and green: HBV-HCC2. The plot indicates the gene expression of all cells in each sample. (**f**) The plot indicates the intensity of *SAA1* or *PLA2G2A* gene expression in all *ALB*-positive cells in each sample.

Serum levels of PLA2G2A in patients with chronic HBV, HBV-induced liver cirrhosis, and HCC (HBV-HCC) were enhanced compared to healthy donors [10]. To confirm whether hepatocytes in HBV-HCC tissues expressed *PLA2G2A*, we performed scRNA-seq on two different HBV-HCC samples. The merged cell population was divided into 14 cell clusters; two main cluster masses in six cancer cells (clusters #6, #10, and #12 from HBV-HCC1 and clusters #1, #4, and #7 from HBV-HCC2), four macrophages (clusters #2, #3, #5, and #10), three adenomas (clusters #9, #11, and a few cells from #2), one *ALB*^high^ hepatocyte (#8), one endothelial cell (#13), one fibroblast (#15), and one *CD8A*^+^ T-cell (#14) (Figure 5a,b). Some macrophages from I-HCA commonly existed with HBV-HCC2 in *CD68*^high^ cluster #3 and *CXCR4*^high^ cluster#5 (dot line in Figure 5b) but not with HBV-HCC1. The feature gene plot and violin of I-HCA markers, *SAA1*, *SAA2*, and *CRP* showed that there were many positive cells in HBV-HCC tissues but *PLA2G2A*^+^ cells were confined to I-HCA (Figure 5c,d). A violin plot comparing *SAA1*, *CRP*, and *PLA2G2A* expression in each sample showed that *SAA1* and *CRP*, which were used as I-HCA markers, were highly expressed in certain HBV-HCC tissues (Figure 5e) but the expression of *PLA2G2A* was low in all cells. However, when the expression levels of *SAA1* and *PLA2G2A* were examined in *ALB*^+^ cells in the respective tissues, *PLA2G2A* was detected only in the I-HCA sample (Figure 5f).

### 3.5. Bulk RNA-seq Analysis for Two Different I-HCA Samples

**Figure 6:**
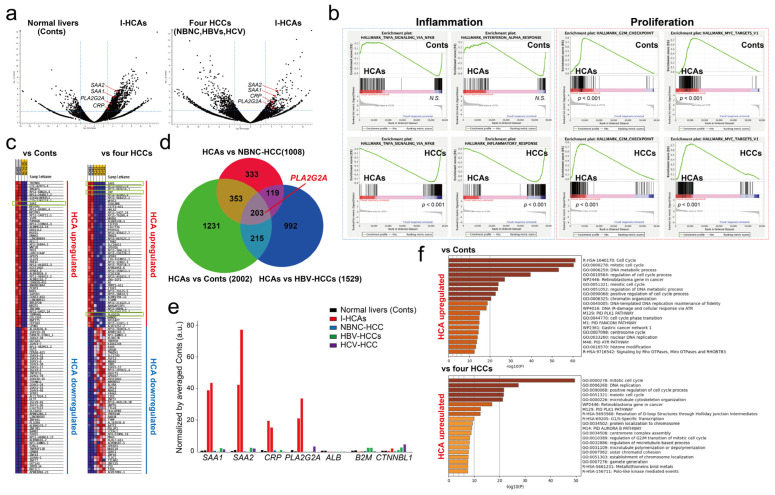
Bulk RNA-seq of two different FFPE-blocked I-HCA samples. (**a**) Visualization of RNA-seq results with a Volcano plot. Left: two normal livers (Conts) vs. two I-HCAs, right: four HCCs vs. two I-HCAs. The dotted blue lines indicate the value of fold changes (±1.5-fold, *x*-axis) and the significance *p*-value (*p* < 0.01, *y*-axis). (**b**) GSEA enrichment plot for “Inflammation” (TNF-α, interferon-α, inflammatory response) and “Proliferation” (G2M checkpoint, MYC targets) with a comparison of two HCAs vs. two Conts or two HCAs vs. four HCCs. The *x*-axis shows genes (vertical black lines) represented in different pathways and the *y*-axis indicates the enrichment score. The color bars at the bottom represent the degree of correlation of the expression of these genes (red to blue through violet, high gene expression to low through middle gene expression). The nominal *p* value is shown under the color bars. Not significant: N.S. The bottom of the plot shows the value of the ranking metric which measures a gene’s correlation with a phenotype. (**c**) Heat map of the gene sets containing all genes found significantly up-regulated or down-regulated (±1.5 fold and *p* value < 0.05) when comparing two I-HCAs vs. two normal livers or two I-HCAs vs. four HCCs. Color ranges from dark red to dark blue representing the highest and lowest expression of a gene, respectively. (**d**) The Venn diagrams of three different conditions; I-HCAs vs. NBNC-HCC, I-HCAs vs. two Conts, and I-HCAs vs. two HBV-HCCs, are shown. The numbers reveal the genes which are considered to be differentially expressed in I-HCA as compared to each condition. For instance, a total of 1008 genes were differently (significance *p* < 0.01 and log_2_ fold change >2.5) expressed in I-HCAs in comparison with the NBNC-HCC sample. The size of the circle reflects the number of genes. (**e**) The graph visualizing the expression levels of selected genes of interest in each sample. The *y*-axis indicates the normalized value (a.u.) by averaged normal livers (Conts). Black bars: normal liver tissues, red bars: I-HCAs, blue bar: NBNC-HCC, green bars: HBV-HCCs, violet bar: HCV-HCC sample. (**f**) Gene ontology analysis by Metascape. The heatmap of enriched terms across the input differently expressed gene lists, colored by *p*-value.

Although confirming that the reproducibility of *PLA2G2A* mRNA expression in fresh I-HCA tissue by scRNA-seq is better, obtaining specimens that had been diagnosed and operated on for I-HCA is challenging. Therefore, we verified the expression of *PLA2G2A* mRNA in other I-HCA tissues which had been embedded in FFPE blocks by RNA-seq. In addition, the total RNA was extracted from two normal liver tissues, one NBNC-HCC, two HBV-HCCs, and one hepatitis C virus (HCV)-derived HCC embedded in FFPE blocks; RNA-seq was then performed. Two volcano plots of two normal livers (Conts) versus (vs) two I-HCAs or four HCCs versus two I-HCAs clearly indicated that *PLA2G2A* was significantly (*p* < 0.05) expressed in I-HCAs (Figure 6a). Gene set enrichment analysis (GSEA) was performed to identify the upregulated and downregulated genes in I-HCA tissues and were enriched in a particular gene set. Representative enriched sets associated with an inflammation such as “TNFα signaling via NFκB” indicated that all the mRNAs in I-HCAs were suppressed as compared to other samples (Figure 6b). In contrast, the enriched set related to cell proliferation such as the “G2M checkpoint” was significantly enhanced in I-HCAs. The heat map of the top 50 features in the two I-HCAs showed that only *SAA1* (rank metric: 4.749) was highly upregulated compared to the two Cont samples when the differential whole gene expression pattern was ordered according to the GSEA rank metric (Figure 6c). In contrast, *SAA1* (rank metric: 4.348), *CRP* (rank metric: 4.071), and *PLA2G2A* (rank metric: 2.496) were significantly upregulated in I-HCAs as compared to the four HCC samples. A detailed analysis using a Venn diagram (set to *p*-values < 0.01 and log_2_ fold changes > 2.5 for each gene) to compare the overlap of differentially expressed genes among tissues indicated that a total of 203 genes were I-HCA-specific and they involved *SAA1*, *SAA2*, *CRP*, and *PLA2G2A* (Figure 6d, Supplementary Data S1). To determine whether *PLA2G2A* was expressed specifically in I-HCAs compared to other liver tissues, genes associated with I-HCA markers were excised from the same dataset and normalized using gene expression levels in two normal liver tissues (Figure 6e). As previously described, both known I-HCA markers and the *PLA2G2A* gene were highly expressed in I-HCA samples. Finally, Metascape [9] was used to objectively determine which gene ontology was upregulated in the I-HCA samples. We analyzed a total of 2846 or 1771 genes upregulated 10-fold higher in two I-HCAs when compared to two Conts or four HCCs groups. This revealed that I-HCAs enhanced expression of gene sets related to cell cycle regulation (Figure 6f), which is consistent with the GSEA enrichment analysis. The 203 genes in a Venn diagram were markedly upregulated in a premalignant lesion I-HCA and were lost in normal and cancerous transformation (Figure 6d). About 15.5% and 13.3% of the genes were already identified as cancer- or disease-related genes and 29.4% had unknown protein functions. About 14% of the genes were long non coding RNAs (lncRNAs), 1.5% were microRNAs, and 2.2% were drug candidate genes for certain FDA-approved diseases. The 123 genes, excluding lncRNAs specifically expressed in I-HCA, were analyzed for signal transduction by Metascape [9] and Reactome [11]. The heatmap showed that cell cycle and DNA repair processes were significantly activated in I-HCA and the 123 genes were correlated with CD34-positive cells or *HSD17B8* regulation (Supplementary Figure S3). Although there was no association between high *HSD17B8* expression and poor prognosis of HCC by the KM-plotter, a total of 122 genes out of 123 genes were correlated with a lower overall survival rate.

## 4. Discussion

In this study, scRNA-seq was employed to compare I-HCA tissue with HCC tissues and normal liver tissue. The analysis revealed that PLA2G2A had a higher specificity for I-HCA compared to SAA1/SAA2 and CRP, which are conventionally used for I-HCA diagnosis. Phospholipase A2 (PLA2) is an enzyme that hydrolyzes cell membrane component phospholipids at the sn-2 position and releases free fatty acids and lysophospholipids [12,13]. PLA2G2A is a secreted protein that belongs to the subfamily of secretory PLA2 and is primarily known for its role in inflammatory and immune responses in various mammalian tissues [13]. The pro- or anti-tumorigenic effects of PLA2G2A appear to be tissue-specific. PLA2G2A is associated with poor survival in patients with esophageal adenocarcinoma [14], glioblastoma [15], rectal cancer [16], and pancreatic ductal adenocarcinoma [17]. In contrast, high PLA2G2A expression suppresses gastric adenocarcinoma and gastric cancer progression [18,19,20,21]. PLA2G2A is suggested to be secreted from cancer cells, eliciting inflammatory responses in the blood circulation. Therefore, increased serum PLA2G2A levels are believed to be reliable biomarkers for diagnosis malignancies [22]. Patients with gastric cancer demonstrated significantly improved survival if the tumors had high PLA2G2A expression because the protein plays a crucial functional role in the suppression of metastasis genes [19]. PLA2G2A mRNA levels are high in primary gastric, colon, and prostate early-stage tumors but low in metastatic and late-stage tumors [18,19]. There is no report about the pro- or anti-tumorigenic effects of PLA2G2A in HCC. According to the TCGA PanCancer Atlas in the liver hepatocellular carcinoma database, high PLA2G2A expression was not correlated with poor prognosis (Supplementary Figure S4).

PLA2G2A expression in normal liver tissue has been discerned at the mRNA level, yet it registers notably low at the protein level [23]. In addition, no correlation was observed between PLA2G2A mRNA expression and survival in HCC using the Kaplan–Meier plotter (Supplementary Figure S4) [24,25]. To the best of our knowledge, only one study has examined the serum levels of PLA2G2A and showed that it was significantly increased in HBV-infected patients [10]. Both PLA2G2A mRNA and protein expression were higher in the HBV-transfected human liver cell line (HepG2.2.15) than in non-HBV-transfected HepG2 cells and the promoter activity of the PLA2G2A gene was predominantly increased in HepG2.2.15. These authors speculate that HBV may upregulate PLA2G2A expression via Wnt/β-catenin signaling. However, our results indicate that the expression of *PLA2G2A* mRNA in HBV-HCC tissues tends to be lower. In addition, *PLA2G2A* expression tended to be higher in the borderline between normal and carcinoma regions (Supplementary Figure S5). The expression of β-catenin (*CTNNBL1*) mRNA in HBV-HCC tissues was not significantly different to that in normal livers (Figure 6e). This is the first report to show that the expression of PLA2G2A in I-HCA tissues was significantly higher than that in normal, NBNC-HCC, HBV, or HCV-HCC tissues. One possibility as to which signaling pathway is upregulated in PLA2G2A expression is the activation of Wnt/β-catenin signaling but it was not enhanced in current two I-HCA tissues by GSEA and Metascape analyses. I-HCA subtypes in which SAA1/SAA2 and CRP are commonly positive have been categorized and three β-catenin-mutated subsets have recently been defined [5,6,26]. The β-catenin mutant activities, which were defined as the different mutated positions and reflected the possibility of tumor progression, are high in more than 70% of HCC tissues. In contrast, the activity in benign HCA was weak (65%), moderate (16%), or high (15%) in 55 HCA tissues [26]. We did not examine whether our I-HCA samples had β-catenin mutations but, even if mutations were present, they did not affect β-catenin expression levels. The correlation between β-catenin mutations and β-catenin mRNA expression levels has not been unclear but the IHC results using anti-PLA2G2A antibody clearly indicated there was no positive reaction in the β-HCA tissue. Our preliminary gene–gene correlation analysis for *PLA2G2A* in hepatocyte cell clusters did not specifically identify co-expressed genes or β-catenin related signaling pathways (Figure 2g). According to the recent studies, β-catenin regulated Lgr5 expression, a G-protein-coupled receptor containing leucine-rich repeats, which is associated with elevated malignancy and unfavorable cancer prognosis [27,28,29]. Lgr5+ cells are not present in normal liver tissue; therefore, the removal of β-catenin in pro-tumorigenic liver tissue such as I-HCA may lead to abnormal immune responses in HCC progression with the suppression of Lgr5 and PLA2G2A. We analyzed other RNA-seq data in normal, nonalcoholic fatty liver disease, and steatohepatitis tissues and it indicated that PLA2G2A mRNA expression was not significantly enhanced in pathological liver tissues with the possibility of being pre-cancerous. In contrast, LGR5 expression increased predominantly with disease progression but it was inconsistent with the CTNNBL1 expression pattern (Supplementary Figure S6). Further studies are warranted on which signaling pathway might regulate PLA2G2A upregulation in I-HCA.

The unique 123 genes, which are expressed at significantly higher levels in I-HCA tissue, were correlated with a poor prognosis of HCC; their expressions were markedly decreased in HCC tissue (Supplementary Figure S3). It is suggested that the genes might regulate the progression of benign to malignant tumors and be new drug candidates with inhibitory effects on cancer progression. For instance, a recently developed drug delivery system, which combines small molecule drugs with two target antibody conjugates, is anticipated to be an effective strategy. Further studies will be warranted to narrow down the unique 123 gene candidates in the future.

## 5. Conclusions

We demonstrated that an increase in PLA2G2A was observed in I-HCA samples and that it was more specifically expressed than the previously known I-HCA markers, SAA1/SAA2 and CRP. Therefore, staining or gene expression analysis of PLA2G2A with known I-HCA markers may help distinguish between HCA and HCC.

## Data Availability

Some data that support the findings of this study are registered in DRA017022 (DRR500608-DRR500611), https://ddbj.nig.ac.jp/search, accessed on 19 December 2023, but other data are restricted from patients’ privacy.

## Supplementary Materials

The following supporting information can be downloaded at https://www.mdpi.com/article/10.3390/cancers16010159/s1. Figure S1: Feature plots of other representative genes of HCA markers in scRNA-seq data. Figure S2: The detailed expression of APOA4 in I-HCA cell clusters. Figure S3: Metascape, Reactome Pathway Database, and Kaplan–Meier (KM) Plotter analysis of I-HCA specifically upregulated genes. Figure S4: KM Plotter related to the effect of the high expression of *PLA2G2A* mRNA on overall survival (OS) of liver cancer. Figure S5: RNA-seq analysis of one HCC and two HCAs. Figure S6: The expression of *PLA2G2A*, *LGR5* and *CTNNBL1* mRNA in healthy, NAFLD and NASH patients [30]. Supplementary Data S1: Data sets of bulk RNA-seq from two HCAs, two normal livers, four HCC samples. Supplementary Data S2: Data sets of bulk RNA-seq from two HCAs, one HCC sample with normal, borderline and HCC; Supplementary Document: Detailed description of Materials and Methods.
