# Supplementary material for: Phospholipase A2 Group IIA Is Associated with Inflammatory Hepatocellular Adenoma
Source: Cancers (Basel). 2023 Dec 28;16(1):159. doi: 10.3390/cancers16010159 (PMC10778238; doi:10.3390/cancers16010159)
Supplement: Supplementary file 1 [file cancers-16-00159-s001.zip › Supplementary Figures.pdf]

Supplementary Figures

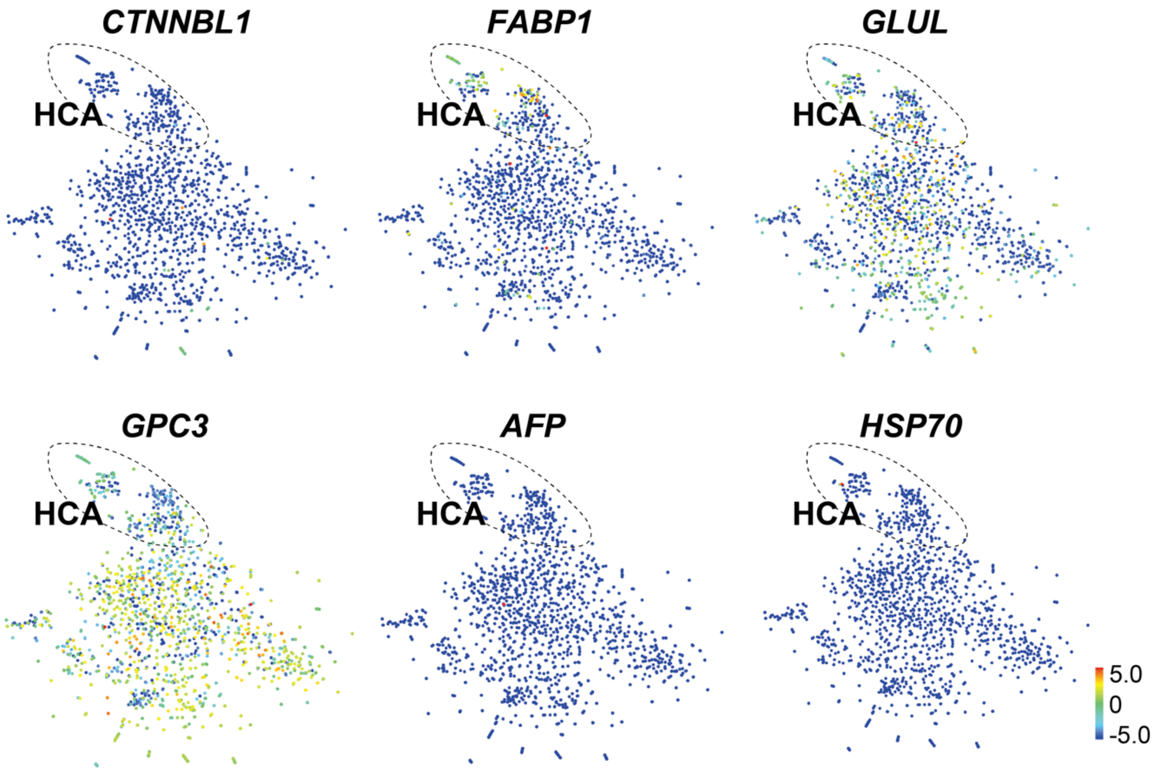

Supplementary Figure S1. Feature plots of other representative genes of HCA markers in scRNA-seq data. Feature plots of six genes, *CTNNBL1*, *FABP1*, *GLUL*, *GCP3*, *AFP* and *HSP70*. The color bar red to blue through yellow reveals the gradual expression intensity differences from high to low through middle.

a

b

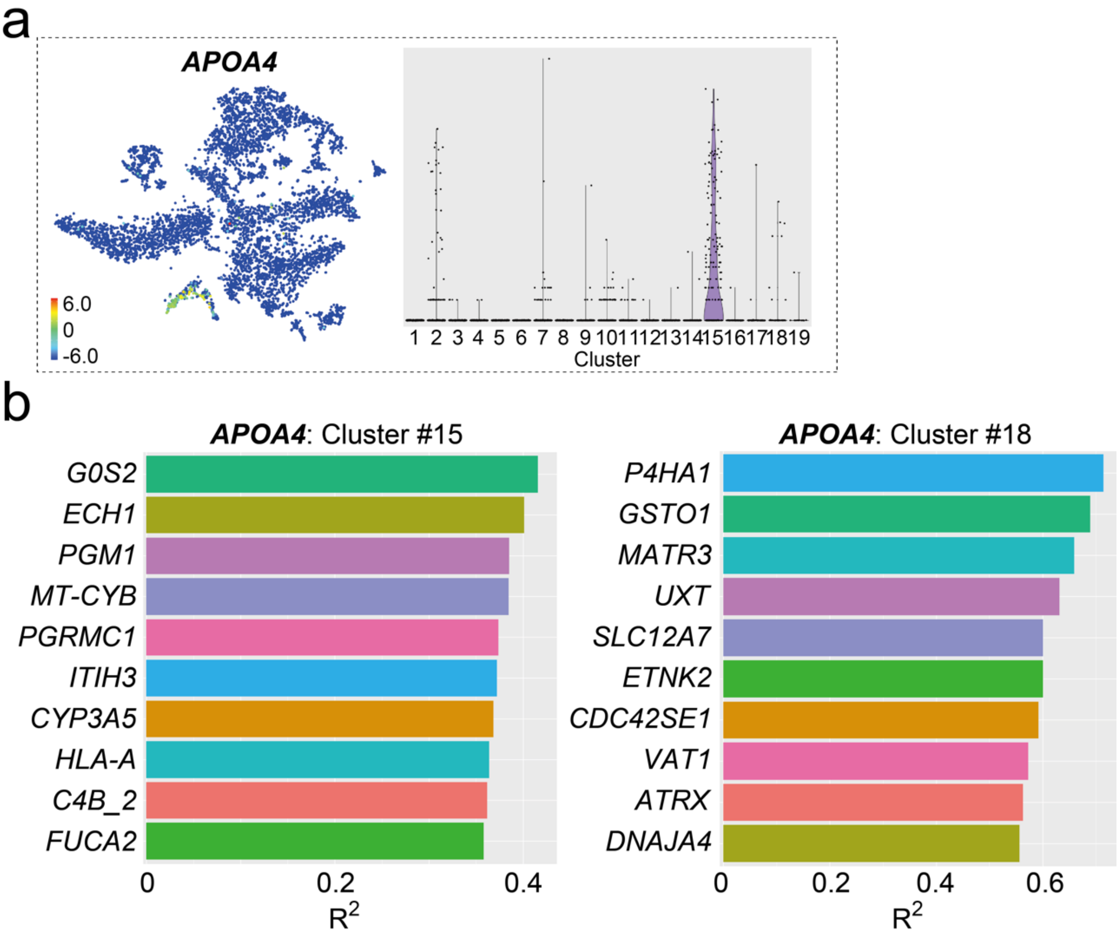

Supplementary Figure S2. The detailed expression of APOA4 in I-HCA cell clusters. (a) The *APOA4* feature plot and violin plot. (b) Gene-gene correlation plots associated with *APOA4* in cluster#15 and cluster#18. X-axis indicates coefficient of determination (R^2).

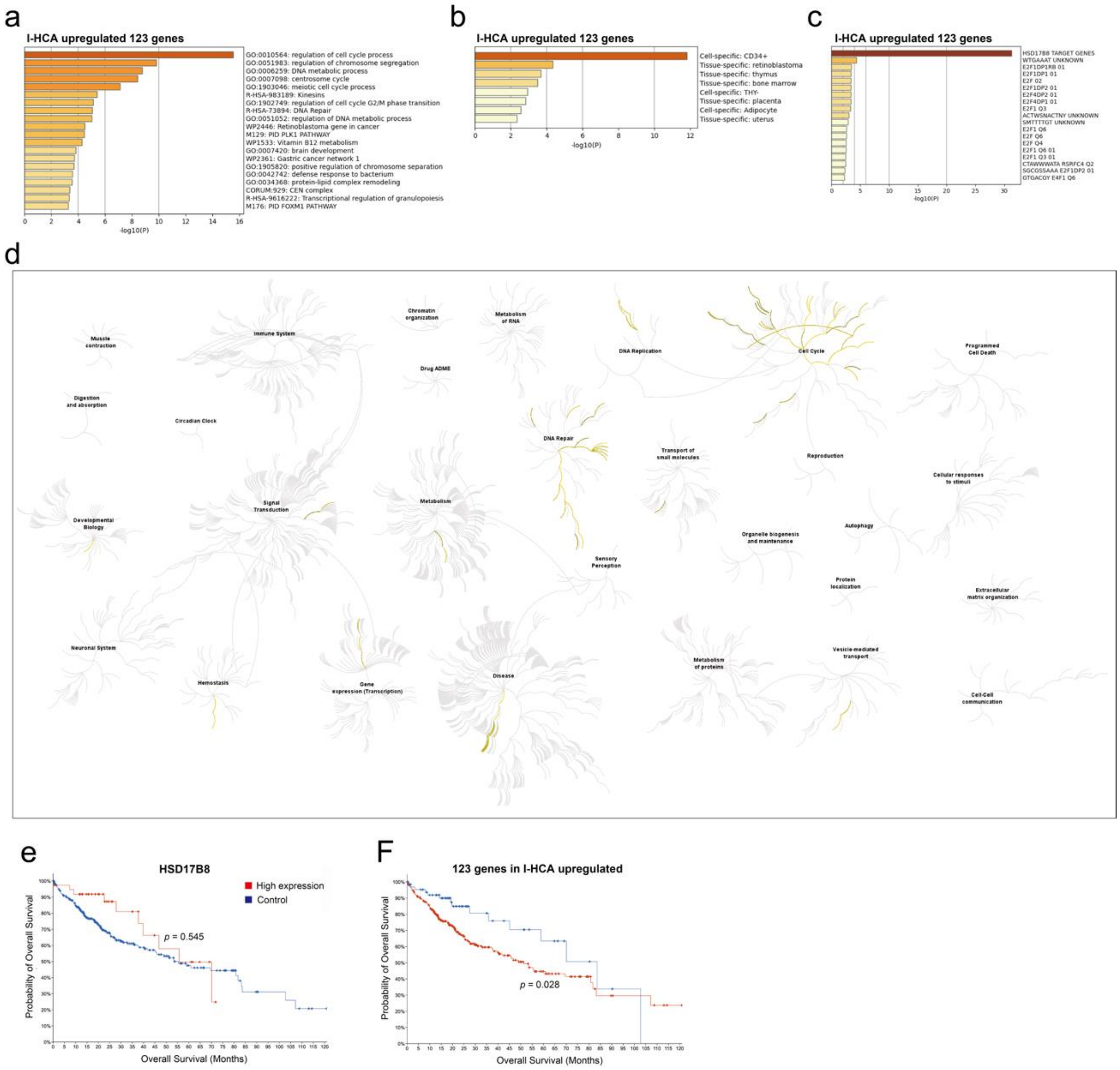

Supplementary Figure S3. Metascape, Reactome Pathway Database and Kaplan-Meier (KM) Plotter analysis of I-HCA specifically upregulated genes. (a) Gene ontology analysis by Metascape [9]. The heatmap of enriched terms (123 genes upregulated in I-HCA) across the input differently expressed gene Top20 lists, colored by *p*-value. (b) Summary of enrichment analysis in PaGenBase. (c) Summary of enrichment analysis in Transcription Factor Targets. (d) Reactome pathway analysis in I-HCA upregulated 123 genes [11]. (e) KM plotter obtained from liver Hepatocellular Carcinoma (TCGA, PanCancer Atlas) in cBio-portal for Cancer genomics. mRNA expression z-scores relative to diplod samples (RNA Seq V2 RSEM, z-score threshold ± 2.0) were selected. Log rank test *p*-value was shown. Red line: Overall survival (OS) of *HSD17B8* mRNA high expression cases. Blue line: OS of control groups. (f) KM plotter of liver Hepatocellular Carcinoma using 123 upregulated genes in I-HCA. Red line: OS of total of 123 mRNA high expression cases. Blue line: OS of control groups. Log rank test *p*-value = 0.028.

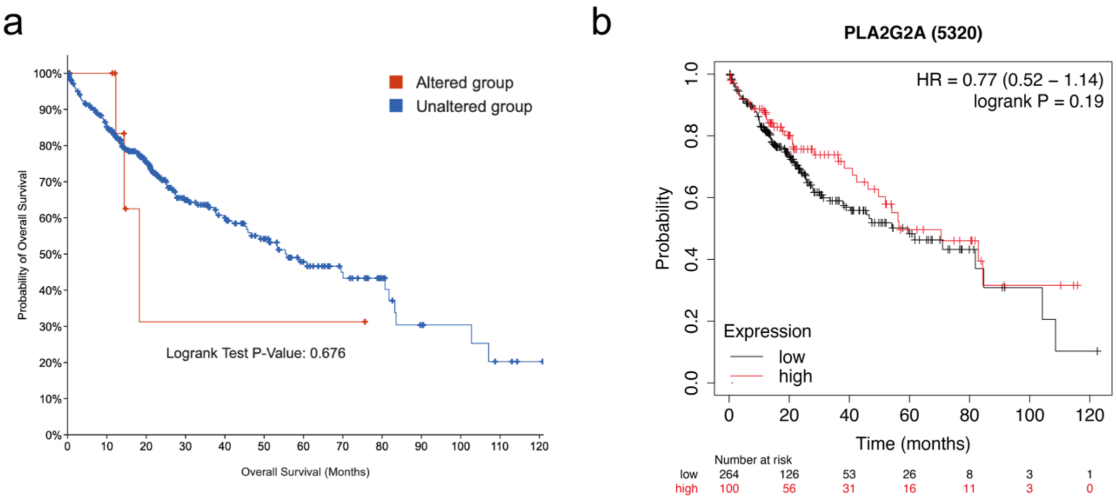

Supplementary Figure S4. KM Plotter related to the high expression of *PLA2G2A* mRNA on overall survival (OS) of liver cancer. (a) KM plotter obtained from liver Hepatocellular Carcinoma (TCGA, PanCancer Atlas) in cBio-portal for Cancer genomics. Samples with PLA2G2A mRNA data (RNA Seq V2, 366 patients, z-scores =±2.0) is shown. Red line: OS of *PLA2G2A* mRNA high expression cases. Blue line: OS of control groups. (b) KM plotter obtained from Liver cancer RNA-seq. The analysis was performed of 364 patients. The red line indicates OS of *PLA2G2A* mRNA high expression cases. Black line: OS of control groups.

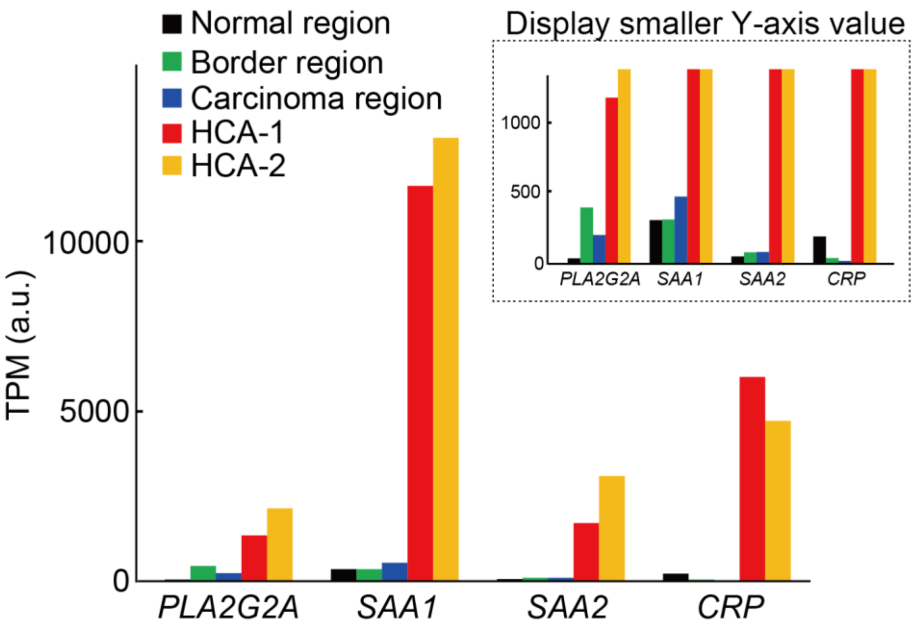

Supplementary Figure S5. RNA-seq analysis of one HCC and two HCAs. The HCC tissue had normal, borderline, and cancerous regions. Total RNA was obtained from each region, and RNA-seq analysis was performed. Y axis: TPM value. Bars indicate raw TMP value. Black bars: normal region, green bars: borderline, blue bars: HCC region, red bars: HCA tissue from patient#1, yellow bars: HCA tissue from patient#2. The raw TPM expression values for PLA2G2A, SAA1, SAA2 and CRP are shown. The right-top panel displays smaller Y-axis TPM value. PLA2G2A expression was relatively higher in border region (green bar in *PLA2G2A*)than in normal (black) or HCC (blue) region.

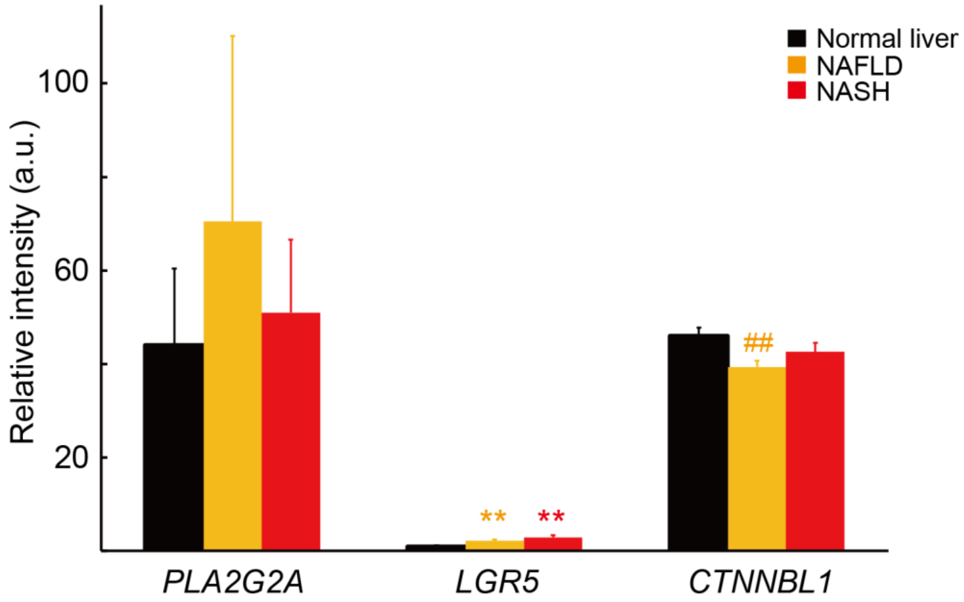

Supplementary Figure S6. The expression of *PLA2G2A*, *LGR5* and *CTNNBL1* mRNA in healthy, NAFLD and NASH patients. Original data was obtained from GSE126848 [30]. The raw count number of genes were normalized by total count. Y axis: Relative intensity (arbitral unit; a.u.). Bars indicate mean ± S.E. Black bars: normal liver (n=14), yellow bars: NAFLD (n=15), red bars: NASH (n=11). There is no significant difference for *PLA2G2A* expression among three different conditions. In contrast, *LGR5* expression increases significantly (**p<0.01) with the progress of liver disease. Inflammatory NAFLD stage, CTNNBL1 mRNA expression is reduced (##p<0.01).
