# Supplementary material for: Phospholipase A2 Group IIA Is Associated with Inflammatory Hepatocellular Adenoma
Source: Cancers (Basel). 2023 Dec 28;16(1):159. doi: 10.3390/cancers16010159 (PMC10778238; doi:10.3390/cancers16010159)
Supplement: Supplementary file 1 [file cancers-16-00159-s001.zip › Supplementary_Document.pdf]

Supplementary Document

Materials and Methods

2.1. Characteristics of the patient

The study included nine patients: three suspected HCA, three with non-B non-C-HCC (NBNC-HCC), two with hepatitis B virus-derived HCC (HBV-HCC), and one with hepatitis C-derived HCC (HCV-HCC). Informed consent was obtained from all patients for participation in the present research and publication of the results. Here, we present only the detailed medical records of patients with HCA whose tissues were analyzed by scRNA-seq. A 33-year-old man with suspected HCC carcinoma and a normal liver background was admitted to the Hospital. He had no smoking history, rarely drank alcohol, had no diabetes, and had a BMI of 22.9. Contrast-enhanced computed tomography (CT) and magnetic resonance imaging (MRI) revealed a mass with a maximum diameter of 60 mm without obvious vascular infiltration. This is atypical of I-HCA. Surgical resection was performed promptly, and the specimens were collected. Subsequent histopathological diagnosis revealed SAA- and CRP-positive tissues.

2.2. Human liver tissue dissociation

The tissues were placed in a culture dish filled with cold Hanks' balanced salt solution (HBSS), and the surrounding excess fibrous connective tissue was removed. The samples were minced using biological tweezers and the pieces were collected in a centrifuge tube with cold HBSS containing 5% fetal bovine serum (FBS). After centrifuged (120 × g, 5 min, 4 ℃), the supernatant was discarded and cold HBSS with 5% FBS was added, then it was centrifuged again. During this processing, Roswell Park Memorial Institute (RPMI) 1640 Medium (Thermo Fisher Scientific, Waltham, MA, USA) with 5% FBS was kept at 37 °C. The supernatant was replaced with the warmed 10 mL RPMI medium and 500 μg of Liberase TH (Roche, Basel, Switzerland), then it was incubated with gentle shaking (300–400 rpm) at 37 ℃ for 30 min. Every 10-15 min, the enzyme solution containing small pieces of tissue was gently diluted by pipetting or using an injection syringe with 18 or 21 G injection needle. Next, 500 μg of DNaseI (Qiagen, D*ü*sseldorf, Germany) was added to the solution with gentle shaking at 37 ℃ for 10 min. When the color of solution changed to light pink, the entire mixture was filtered using 100 μm Cell Strainer with gentle mashing using a piston for an injection cylinder. Next, 10-15 mL of cold RPMI medium with 5% FBS was added and centrifuged (330 × g, 5 min, 4°C), and the supernatant was completely removed. If the cell pellet volume was large, it was divided into different tubes. The supernatant was replaced to 4.8 mL of fresh cold RPMI medium in each tube.

A 100% Percoll solution (Cytiva, Marlborough, MS, U.S.A.) was prepared using 10 mL Percoll and 1 mL cold 10 × HBSS. 1.2 mL of 100% Percoll and 800 μL of HBSS was mixed gently (60% Percoll), then 2 mL of 60% Percoll solution was added to the new 15 mL of centrifuged tube. Next, 1.2 mL of cold 100% Percoll was added to 4.8 mL of cell solution (20% Percoll), and 6 mL of 20% Percoll solution with cells was added to the 60% Percoll solution while inclining the tube. After centrifuged (1,000 × g, 20 min, 20 ℃; deacceleration mode was slow), an upper layer of 20% Percoll solution was removed, an intermediate layer with target cells was carefully moved to a new 15 mL centrifuged tube, and cold HBSS was added to 10 mL. Subsequently, the tube was centrifuged (120 × g, 3 min, 4 ℃); the supernatant was replaced to 10 mL of fresh cold PBS and centrifuged again. This washing process was performed at least 2 times to completely remove the Percoll. Next, the cell pellet was kept in ice, and 1 mL of cold 0.8% ammonium chloride solution was added for 2 min. This washing

process was repeated 2 times. Finally, 1 mL of cold PBS was added, and the isolated cell solution was filtered using 40 µm Flowmi™ Tip Strainers (Bel-Art-SP Scienceware, Wayne, NJ, USA).

2.3. Single-cell RNA-sequencing (scRNA-seq)

We used an Nx1-seq (next-generation 1-cell sequencing) device for scRNA-seq [7]. The major components of Nx1-seq are barcode beads and a microwell composed of polydimethylsiloxane (PDMS). The PDSM microwell plate was placed in an oxygen plasma chamber (YHS-R, SAKIGAKE-Semiconductor Co., Kyoto, Japan) for 6 min for the hydrophilic process, because PDMS is a hydrophobic material. After blocking using 1% bovine serum albumin (BSA) for 30 min, 1 mL of PBS with ~ 5×10^5 barcode beads was added to the microwell plate for at least 30 min with gentle agitation. The protocol for preparing barcoded beads was slightly modified from that described in the instruction manual for the GS Junior Titanium emPCR Kit (Lib-B) (Roche) [8]. After single-cell isolation, approximately 2×10^5 cells mixed with 3.7 mL cold PBS were added to the PDMS microwell plate and covered without bubbles. The microwell plate was placed on ice for 10-15 min, to allow the cells to settle in the microwell due to gravity. Approximately 5% of the microwells were filled with single cells according to the Poisson distribution. The solution was removed from the microwell plate and 1 mL of fresh cold PBS was gently applied. The solution was completely removed and 1 mL of fresh cold PBS was added again. The washing process was repeated 3-4 times until the cells in the space between each microwell were removed. If cells exist, the mRNA dissolved by the cell lysis buffer will affect the neighboring barcode beads in the microwell.

The reagent composition of 1 mL of cell lysis buffer was: *N*-Lauroylsarcosine sodium salt (2 mg), 1M Tris-HCl pH 7.5 (200 µL), 0.5M EDTA pH 8.0 (40 µL), deionized water (750 µL), 1M dithiothreitol solution (DTT) (50 µL). The PBS was removed, and 1 mL of cell lysis buffer was gently added along the corner of the microwell. The cell lysis buffer was removed and 1.5 mL of washing buffer based on the cell lysis buffer (washing buffer:200 mM Tris-HCl pH 7.5, 20 mM EDTA, 50 mM DTT, 0.2% *N*-lauroylsarcosine sodium salt, 2% Ficoll) was added. A surgical blade was used to add it to a 1.5 mL low binding protein tube filled with 1 mL of 6X saline sodium citrate (SSC) buffer. The tubes were centrifuged at the maximum rpm for less than 20 s, turned in the opposite direction, and centrifuged again until barcode beads were observed at the bottom of the tube. After removing SSC buffer carefully not include bar-code beads, fresh 1 mL of 1X first strand SSIV buffer (SuperScript™ IV Reverse Transcriptase, Thermo) was added to the tube. After removed the solution, the reagent for reverse transcriptase (RT buffer; 6.6 µL of 5X SSIV buffer, 6.6 µL of 5 M Betain solution, 1.98 µL of 100 M $MgCl_2$, 1.76 µL of 0.1M DTT, 1.76 µL SSIV, 1.42 µL of Recombinant RNase Inhibitor (Takara BIO Inc., Shiga, Japan), 1.32 µl of 25 mM dNTP, 14.2 µL of deionized water) was added, and the mixture was incubated at 52 °C for 25 min with stirring at 800 rpm. After that, the RT buffer was removed, 200 µL of ExoI buffer (20 µL of 10X ExoI Reaction Buffer (New England Biolabs [NEB], Ipswich, MS U.S.A), 10 µL of ExoI, 170 µL of deionized water) added the mixture was incubated at 37 °C for 30-40 min with stirring by 800 rpm. After the reaction, the tube was kept at 80 °C for 20 min, then placed on ice for 10 min. The solution was replaced with 1000 µL Tris-HCl, EDTA with Tween20 (TE/TW) buffer (10 mM Tris pH8.0, 1 mM EDTA pH8.0, 0.01% Tween20).

After removing the supernatant, RNase H buffer (4 µL of 5X FS buffer, µL of 0.1 M DTT, 1.6 U of RNase H [Thermo], 14.2 µL of deionized water) was added, and the mixture was incubated at 37 °C for 20 min. The supernatant was replaced to TE/TW buffer, and then TdT

mixture (4 μL of 5X TdT buffer [Takara], 2 μL of BSA, 4 U of TdT, 0.6 μL of 100 μM dATP [Roche], 12.4 μL of deionized water) was added. The mixture was incubated at 37 °C for 90 seconds with gentle tapping, then immediately put on ice with 10 μL of 0.5 M EDTA and 200 μL of TE/TW buffer. The tube was kept at 65 °C for 10 min to stop the enzyme activity. After washing the beads by TE/TW at least three times, beads with 20 μL of second-strand synthesis mixture (10 μL of 1X KAPA Hifi ReadyMix [KAPA Biosystems, Wilmington, MA U.S.A], 1 μL of 10 mM 5′tagging primer [5′-CTATGCGCCTTGCCAGCCCGCATCTGT23 TTTTTTTTTTTTTTTTTTTTTTTVN-3′], 9 mL of deionized water) were transferred into new 8-strip tubes, and second-strand synthesis was performed (95 °C for 3 min, 44 °C for 2 min, 72 °C for 7 min, then 4 °C). After removing the solution, 200 μL of TE/TW buffer was added and washed at least twice. Polymerase chain reaction (PCR) reaction mixture (25 μL of 1X KAPA Hifi ReadyMix, 1.75 μL of 10 mM 3′ primer [5′-Biotin-CGTATCGCCTCCCTCGC GCCATCAGAC-3′], 1.75 μL of 10 mM 5′ primer [5′-Biotin-ATGCGCCTTGCCAGCCCGCA GTCTGT-3′], 21.5 μL of deionized water) was added to the 8-strip tubes, and the PCR mixture was amplified using a thermal cycler (95 °C for 1 min; 16 cycles of 98 °C for 10 s, 65 °C for 30 s, 68 °C for 3 min; 72 °C for 10 min; then 4 °C). The PCR products were purified using 30 μL of AMPure XP Beads (Agilent Technologies, Inc., Santa Clara, CA, U.S.A) and eluted with 10 μL of 10 mM Tris-HCl (pH 8.0) (elution buffer; EB). To check the size of the PCR product, 1 μL of 6X loading buffer (Takara) and 1.5 μL of Midori Green Direct (Nippon Genetics, Osaka, Japan) were added, and cDNA was loaded onto 1-1.5% Agarose gels (Invitrogen, Waltham,MS U.S.A) and run at 100 V for 25 min. The gel containing cDNA between 500 bp and 3 kbp was cut and eluted using a QIAquick gel extraction kit (Qiagen) in 20 mL of EB with 30 mL of TE buffer pH 8.0.

A total of 50 μL of the solution was treated using a Covaris Focused-ultrasonicator M220 (M&S Instruments Inc., Osaka, Japan) with peak power of 50 W, duty factor of 20%, cycles/burst 200 counts, and duration of 120 s. The fragmented cDNA was purified using 45 μL AMPure XP Beads and eluted with 28 μL EB. To perform end-repair and dA tailing, 3.25 μL of NEBNext Ultra II End Prepction Buffer (NEB) and 1.5 μL of NEBNext Ultra II End Prep Enzyme Mix were added and the mixture was incubated at 20 °C for 30 min, 65 °C for 30 min, then 4 °C. AMPure XP Beads (32.5 μL) were added and eluted with 14 μL of EB. 1 μL of dsDNA Solexa adaptor [(200 ng/mL) prepared using two oligos (10 μL of 100 mM 5′-GTGACTGGAGTTCAGACGTGTGCTCTTCCGATCTT-3′ and 10 μL of 100 mM 5′-Phosphorylation-AGATCGGAAGAGCACACGTCTGAACTCCAGTCAC-Amino modifier-3′ , 1.56 μL of TE buffer pH 8.0) was mixed and annealed using a thermal cycler (95 °C for 2 min, 65 °C for 10 seconds, 37 °C for 10 min, 20 °C for 20 min, then 4 °C), then deionized water (43.1 μL) was added] was added to 14 μL of EB, and the mixture was incubated at 55 °C for 5 min, then kept at 20 °C for 10 min. 10 min later, 10 μL of Ligation high Ver.2 (Takara) was added to the tube. The mixture was then incubated at 16 °C for 1 h. Next, 25 μL of AMPure XP Beads were added and eluted with 21.5 μL of EB. Finally, 28.5 μL of index PCR mixture (25 μL of 1X KAPA Hifi ReadyMix [KAPA Biosystems], 1.5 μL of 10 mM P5-adaptor primer [5′-AATGATACGGCGACCACCGAGATCTACACGCCTGTCCGCGGCGTATCGCCTCCC TC GCGCCATCAG*A*C-3′], 1.5 μL of 10 mM P7-index primers based on Illumina Tru-seq RNA Library Prep Kit v2 [Illumina Inc., San Diego, CA U.S.A.] [5′-CAAGCAGAAGACGGCATACGAGAT-index-GTGACTGGAGTTC-3′]) was added and the cDNA was amplified using a thermal cycler (95 °C for 45 s; 12 cycles of 98 °C for 15 sec, 65 °C for 15 s, 72 °C for 30 s; 72 °C for 3 min; then 4 °C). The PCR products were purified with 50 μL AMPure XP Beads and eluted with 30 μL EB.

The quality of the sequencing libraries was evaluated using an Agilent 4200 TapeStation (Agilent Technologies) and the concentration was measured using a Qubit Fluorometer (Thermo). The number of libraries was estimated using a KAPA Library Quantification Kit (Roche). The average length of the I-HCA library was 466 bp. High-throughput sequencing was performed on samples with a 20% PhiX control using the MiniSeq High Output Kit (Illumina, 150 cycles pair-end, 25/125 or 75/75 cycles) or NextSeq 500/550 High Output Kit v2.5 (Illumina, 150 cycles pair-end, 25/125 or 75/75 cycles). Custom sequence read 1 seq primer (5′-GCCTGTCCGCGGCGTATCGCCTCCCTCGCGCCATCAGAC-3′) was used. Pair-end FASTQ files were mapped and annotated using bowtie2 software and Perl custom scripts [1]. Read 1 contained the barcode sequence (12 N) and the primer sequence following it; the read 2 sequence was from each cell mRNA. Barcode sequences were extracted from read 1 fastq file. The read 2 sequences were aligned against RefSeq mRNA (ftp://ftp.ncbi.nih.gov/refseq/H_sapiens/mRNA_Prot/) as a reference sequence using bowtie 2 software. After mapping, the barcode was linked to its paired read 2 alignment data and the genes were counted for each barcode. Cell clustering was performed by t-distributed Stochastic Neighbor Embedding (tSNE) analysis using Seurat v2.3.0, on R-3.6.3.

2.4. Immunohistochemistry (IHC)

In accordance with standard methods, formalin-fixed paraffin-embedded (FFPE) tissues were prepared by fixing fresh tissues using 4% paraformaldehyde at 4 °C and embedding them in paraffin. Tissue sections (4 μm thick) were cut, dewaxed, and rehydrated using xylene and graded alcohol. Hematoxylin and eosin (H&E) staining was performed. The sections were then inactivated by treating them with an antigen activator (citric acid pH 6.0) for 20 min at 95 °C, and they were incubated with anti-human PLA2G2A polyclonal antibody (1:200 dilution, PA5-102403, Thermo) or anti-human SAA1+SAA2 (1:150 dilution, PA5-102456, Invitrogen) overnight at 4 °C. Thereafter, the sections were treated with rabbit anti-IgG antibodies for 30 min at 20 °C and visualized following treatment with 3,3-diaminobenzidine for 10 min at 20 °C. Subsequently, the sections were counterstained with hematoxylin. All stained sections were examined under a fluorescence microscope (BZ-X710; KEYENCE, Osaka, Japan).

2.5. RNA sequencing (RNA-seq)

Some tissue sections (10 μm thick) from the FFPE block in each sample were cut and put into 1.5 ml tube, and total RNA was obtained using NucleoSpin total RNA FFPE kit (Marcherey-Nagel GmbH & Co. KG, Düren, Germany) according to the manufacturer's instructions. In brief, the paraffin dissolver solution was treated to the tube for 5 min at 56 °C and with proteinase K for 90 min. After DNase treatment, approximately 100–400 ng/μL of high-purity RNA was eluted and stored at −80 °C for further analysis. RNA quality was evaluated using an Agilent 4200 TapeStation (Agilent Technologies) and RNA concentration was measured using a Qubit Fluorometer (Thermo). A total of 1000–3500 ng of RNA from each sample was used, and libraries for sequencing were constructed using TruSeq Stranded mRNA (Illumina) according to the manufacturer's protocol. The number of libraries was estimated using a KAPA Library Quantification Kit (Roche). The average library size was 300 base pairs. High-throughput sequencing of the samples was performed using a NextSeq 500/550 High Output Kit v2.5 (Illumina, 75 cycles pair-end, 40/40 cycles). The average number of sequences reads per sample was 26,807,076. The bulk RNA-Seq results were analyzed using the CLC Genomics Workbench Version 12.0.2 (Filgen Inc., Nagoya, Japan). The raw transcript per million (TPM) data obtained from RNA-seq are provided in the

Supplementary Data. Target gene sets were analyzed using the Gene Ontology enrichment analysis tool Metascape [9].
